# Assessing transporter‐mediated rifampin–linezolid interaction using physiologically‐based pharmacokinetic modelling

**DOI:** 10.1002/bcp.70443

**Published:** 2026-01-26

**Authors:** Hoang Dat Nguyen, Vinh Hoa Pham, Richard M. Hoglund, Joel Tarning, Junjie Ding

**Affiliations:** ^1^ Master of Science Program in Biopharmaceutical Sciences, Department of Biochemistry, Faculty of Pharmacy Mahidol University Bangkok Thailand; ^2^ Department of Pharmacology and Pharmacogenomics Research Center Inje University, College of Medicine Busan Republic of Korea; ^3^ Mahidol Oxford Tropical Medicine Research Unit, Faculty of Tropical Medicine Mahidol University Bangkok Thailand; ^4^ Centre for Tropical Medicine and Global Health, Nuffield Department of Clinical Medicine University of Oxford Oxford UK; ^5^ Infectious Diseases Data Observatory University of Oxford Oxford UK

**Keywords:** drug–drug interaction, linezolid, physiologically‐based pharmacokinetic model, rifampin, transporter

## Abstract

**Aims:**

The study aims to develop a physiologically‐based pharmacokinetic (PBPK) model to quantitatively evaluate the role of ATP‐binding cassette sub‐family B member 1 (*ABCB1*) and ATP‐binding cassette super‐family G member 2 (*ABCG2*) in the drug–drug interaction (DDI) between rifampin and linezolid and to predict the impact of high‐dose rifampin on linezolid pharmacokinetics (PK).

**Methods:**

We developed a PBPK model of linezolid and verified this using published clinical PK data. The built‐in PK‐SIM PBPK model for rifampin was used as a perpetrator model, which incorporate *ABCB1* and *ABCG2* transporter activity, along with inhibition and induction kinetic parameters. Using the developed PBPK models, linezolid PK was predicted when co‐administered with rifampin and verified using published data. Based on the developed DDI model, linezolid exposure when co‐administered with high‐dose rifampin at steady state was predicted.

**Results:**

The developed linezolid PBPK model had acceptable predictive performance for 36 different PK arms from 13 individual clinical studies. The PBPK‐predicted DDI effect of standard dose rifampin on linezolid, with AUC and *C*_*max*_ ratios of 0.77 and 0.87, respectively, aligned well with observed DDI ratio. PBPK simulations indicated that both *ABCG2 and ABCB1* contributed to the DDI between linezolid and rifampin, with *ABCB1* playing the major role in the interaction. Increasing the daily dose of rifampin from 10 mg/kg to 20–40 mg/kg resulted in a similar linezolid exposure.

**Conclusions:**

Our study suggested that *ABCB1* is the primary transporter responsible for the interaction between rifampin and linezolid. The DDI effect of high‐dose rifampin on linezolid plasma exposure is similar to that of standard‐dose rifampin.

**What is already known about this subject?**

- Rifampin has been shown to significantly reduce the plasma exposure of linezolid in clinical studies, but the primary transporter mediating this interaction remains controversial.
- High dose rifampin (>20 mg/kg) plus linezolid have shown potential in the treatment of tuberculous meningitis, but the extent of their drug‐drug interaction must be evaluated to ensure both safety and efficacy.
- Physiologically‐based pharmacokinetic (PBPK) modeling has emerged as a powerful tool for evaluating the roles of enzymes and transporters in drug‐drug interactions and for simulating these interactions under various clinical scenarios.

**What this study adds?**

- A PBPK model of linezolid was developed and extensively validated using the largest number of studies to date, demonstrating excellent predictive performance.
- Both *ABCB1* and *ABCG2* were identified to contribute to the drug‐drug interaction between rifampin and linezolid, with *ABCB1* playing the major role in the interaction.
- At high dose of rifampin (20–40 mg/kg), the model predicted a similar reduction in linezolid plasma exposure compared to that observed with the standard 10 mg/kg rifampin dosing regimen.

## 1 INTRODUCTION

Tuberculosis (TB) remains the leading cause of death among infectious diseases with around 10 million newly reported cases each year.^1^ A major obstacle to achieving the goals of the end TB strategy is the emergence of drug‐resistant TB. Globally, 3.4% of newly diagnosed TB patients and 18% of those with prior TB treatment were found to have rifampicin‐resistant or multidrug‐resistant (MDR) TB.^2^ MDR‐TB requires intensive treatment, often involving a longer duration with additional medications.

Linezolid, an antibiotic of the oxazolidinone class, is effective against a wide range of gram‐positive bacterial infections, including MDR tuberculosis.^3^

Linezolid was re‐classified as a Group A drug by the World Health Organization (WHO) for treatment of MDR‐TB and extensively drug‐resistant tuberculosis (XDR‐TB).^4^ Linezolid shows desirable PK properties, with very high oral bioavailability and low plasma protein binding. Linezolid undergoes predominantly non‐renal elimination, with renal elimination occurring only to a small extent.^5^ Studies suggest that linezolid is a substrate of the transporters *ABCB1* and *ABCG2*,^6^ and transporter‐mediated drug–drug interactions (DDIs) cannot be ruled out when co‐medicated with a transporter inducer or inhibitor. A clinical DDI study showed that clarithromycin, a strong inhibitor of *ABCB1*, increased linezolid AUC by 81% in TB patients.^7^, ^8^ Co‐administration of levothyroxine, a *ABCB1* inducer, significantly reduced linezolid exposure.^9^, ^10^

Rifampin is a cornerstone drug in first‐line TB treatment and has been shown to exhibit a synergistic therapeutic effect when combined with linezolid.^11^ Rifampin is a well‐known inducer of *ABCB1*, but can also act as an inhibitor at high concentration.^12^ Additionally, rifampin inhibits *ABCG2* activity^13^ while inducing breast cancer resistance protein (BCRP */ABCG2*) expression, increasing its levels by 1.18 to 2.7‐folds.^14^, ^15^, ^16^ A clinical DDI study showed that standard‐dose of rifampin (10 mg/kg) significantly reduced linezolid plasma exposure, with reported decreases of 35%–37% in AUC^17^ and 20% in *C*_*max*_.^18^ We hypothesize that the reported PK DDI between rifampin and linezolid is mediated primarily through *ABCB1*, with additional contribution from *ABCG2*.

The combination of rifampicin and linezolid has been shown to be effective against methicillin‐resistant *Staphylococcus aureus* (MRSA) in preclinical vivo models and clinical studies^19^, ^20^, ^21^.^22^ Recently, the addition of linezolid to the standard treatment regimen for tuberculous meningitis (TBM) has been evaluated in a Phase 2 clinical trial and demonstrated a favourable safety profile.^23^ Additionally, Davis et al. reported that high‐dose rifampicin and adjunctive linezolid can safely be added to the standard of care in HIV‐associated TBM.^24^ Currently, a large Phase 3 clinical study is being conducted to further assess the efficacy of high‐dose rifampin plus linezolid in the treatment of TBM.^25^ However, DDI effect of rifampin–linezolid was based on the standard dose of rifampin, which limits the ability to predict DDI effects at higher dose levels, as rifampin exhibit concentration‐dependent *ABCB1* induction or inhibition.^26^ Addressing this gap requires physiologically‐based pharmacokinetic (PBPK) modelling, a mechanistic in silico approach that quantitatively predicts DDIs according to transporter‐kinetic parameters.^27^, ^28^

In this study, we aimed to utilize a PBPK modelling approach to quantitatively assess the roles of *ABCB1* and *ABCG2* in the DDI between rifampin and linezolid, providing deeper mechanistic insights into this interaction, and predict linezolid PK in the context of standard and high‐dose rifampin administration to inform clinical practice.

## 2 METHODS

### 2.1 Software

The PBPK model for linezolid was developed using the PK‐Sim® software (version 11.3). Visualization of PBPK results and PK exposure calculations were performed using R (version 4.0.2, The R Foundation for Statistical Computing) within RStudio (version 4.2.3, RStudio, Inc., Boston, MA, USA).

### 2.2 Clinical PK data

Clinical PK data of linezolid after monotherapy in healthy populations were collected using a systematic search of the PubMed database. This search was conducted using the keywords ‘linezolid pharmacokinetics healthy’ and ‘linezolid bioavailability’ to identify studies published between January 2000 and December 2024. Studies were included if they reported linezolid PK profiles and parameters under fasted conditions (following single or multiple doses) and provided clinical information, including drug dose and demographic data (age, weight and gender). Studies that were inaccessible or published in languages other than English were excluded.

In total, 13 clinical PK studies comprising of 36 distinct study arms were included for model development and evaluation (Table S1). Of these 36 study arms, 31 reported full PK profiles while the other 5 contained only sparse data. The dose range across these studies varied from 375 to 600 mg, administered as single or multiple doses.

**FIGURE 1:**
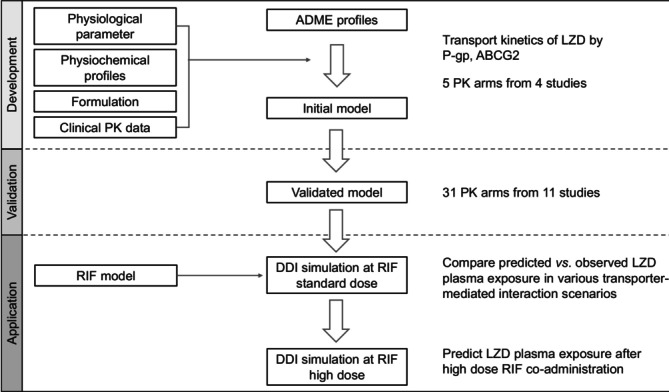
Workflow for PBPK model development and validation of linezolid and its application in predicting drug–drug interaction with rifampin in healthy volunteers.

The selection of study arms for the training dataset was determined through assessment of study relevance and representativeness. In brief, the training dataset included 4 clinical studies (with a total of 5 PK arms), representing different clinical scenarios, such as different dose amounts (e.g., 375 or 625 mg), regimens (single or multiple administrations) and routes of administration (IV and oral). The remaining 31 study arms from 11 studies were used as the validation dataset (Figure 1).

### 2.3 PBPK modelling workflow

The model development and validation process adhered to established best practices for PBPK modelling, as outlined in relevant guidelines.^27^

The PBPK modelling workflow for the rifampin–linezolid DDI is shown in Figure 1. It included the development of a PBPK model for the victim (linezolid), the perpetrator (rifampin) and the DDI model.

#### 2.3.1 Linezolid PBPK model

Physiological parameters were obtained from the built‐in PK‐SIM population, and drug‐specific properties (e.g., transporter kinetic parameters) were systematically gathered from literature. The model was developed based on intravenous administration of linezolid and calibrated with observed data associated with intravenous administration. Then, the model was extended to include oral absorption and further optimized using observed oral PK data. During model development, a local sensitivity analysis was conducted to identify the parameters with the greatest influence on PK exposure. The prioritization of parameter optimization followed a general guidance outlined in a published tutorial on PBPK model development.^27^ Typically, during IV PBPK model development, distribution‐related parameters (e.g., lipophilicity and partition coefficient) are optimized first, followed by clearance‐related parameters (e.g., *K*_*cat*_). Once the IV PBPK model was established and refined, absorption‐related parameters (e.g., permeability) were optimized.

#### 2.3.2 Rifampin PBPK model

A built‐in validated PK‐SIM PBPK model was adapted (https://github.com/Open-Systems-Pharmacology/OSP-PBPK-Model-adliationLibrary/tree/master/Rifampicin). In brief, the rifampin PBPK model was previously developed and verified using data from 11 clinical PK studies, including 18 distinct study arms. Rifampin is a substrate of *AADAC*, *OATP1B1* and *ABCB1* and the PBPK model incorporated the auto‐induction of *ABCB1*, *OATP1B1 and AADAC* transporters, with an *EC*_50_ value of 0.34 μmol/L for all transporters and an *E*_*max*_ value of 2.5, 0.383 and 0.985, respectively.^27^ The inhibition of *ABCG2* and *ABCB1* by rifampin was described using inhibition constant (*K*_*i*_) values of 14 and 9.1 μmol/L, respectively.^29^

The constructed DDI model combined the developed rifampin and linezolid compound models, with associated transporter kinetic parameters. Based on the developed DDI model, drug exposure to linezolid could be predicted when co‐administered with standard dose and high‐dose of rifampin.

### 2.4 Key parameters in linezolid PBPK model building

According to the linezolid drug label, the average total plasma clearance following intravenous administration is approximately 100–200 mL/min, with hepatic clearance estimated at 70–150 mL/min and renal clearance contributing to around 30–50 mL/min.^5^ Given that the specific metabolic pathways responsible for the conversion of linezolid to its primary metabolites (PNU‐142300 and PNU‐142586) remain unclear,^5^ we assumed here that non‐renal clearance primarily occurs via hepatic metabolism and used total hepatic clearance to describe this process.

Total plasma hepatic clearance was then optimized to achieve a similar total plasma clearance (average 100 mL/min). Linezolid has been reported to exhibit slightly reduced clearance at steady state compared with single‐dose administration.^30^ This reduction in hepatic clearance may be associated with a time‐dependent decrease in mitochondrial respiratory‐chain enzyme activity, which in turn reduces cellular NADPH.^31^ To accurately capture this PK characteristics after repeated dosing, the total hepatic clearance was optimized for multiple dose regimen by reducing the hepatic CL by 15% at steady state.

The clearance via renal route was described by incorporating the transporters *ABCB1* and *ABCG2*, both of which are involved in linezolid efflux and are highly expressed in the intestine, liver and kidneys. Transporter assays showed a protein‐normalized intrinsic efflux clearances (*CL*_*int*_) of 2.1 μL/min/mg for *ABCB1* and 16 μL/min/mg for *ABCG2*.^6^ The *K*_*m*_ values for *ABCB1* and *ABCG2* were not available. We therefore assumed that *K*_*m*_ was equal to the incubated concentration of 100 μM, while the *V*_*max*_ was calculated according to Equation (1).

(1) $$V_{\max}={\mathrm{CL}}_{\mathrm{int}}\times K_{m}$$

The maximum catalytic activity (*K*_*cat*_) was determined (Equation 2) as described by PK‐Sim (https://docs.open‐systems‐pharmacology.org/v9/working‐with‐pk‐sim/pk‐sim‐documentation/pk‐sim‐expression‐data)

(2) $$K_{\mathrm{cat}}=\frac{V_{\max}}{\text{Transporter abundance}}$$

For *ABCB1*, with a protein‐normalized *CL*_*int*_ of 2.1 μL/min/mg, and transporter abundance of 10.3 pmol/mg,^6^ the *K*_*cat*_ was calculated to be 200 min^−1^. For *ABCG2*, with a protein‐normalized *CL*_*int*_ of 16 μL/min/mg, and transporter abundance of 13.18 pmol/mg,^6^ the *K*_*cat*_ was calculated to be 120 min^−1^. The *K*_*m*_ and *K*_*cat*_ parameters were further optimized during the model development.

One study^32^ reported a mean recovery of total radioactivity of linezolid in faeces of 9.9% ± 3.4%.However, it was unclear whether it represented unchanged drug or metabolites. A recent study showed that the majority of linezolid recovery in faeces was due to a metabolite and that parent drug is not excreted in the feces.^33^ We therefore concluded that linezolid is not excreted via the gallbladder. To describe this process in the PBPK model, the expression of *ABCG2* and *ABCB1* in the liver was removed accordingly, resulting in a predicted urine excretion of 30%–40% of the parent drug. This prediction was similar with the observed urinary excretion of 30%–35%, reported in a previous study.^34^

### 2.5 DDI model

The developed linezolid PBPK model and built‐in rifampin model was used to predict linezolid exposure when co‐administered with rifampin. The rifampin model incorporated the inhibition of *ABCB1* and *ABCG2*, along with the induction of *ABCB1*, which mediated the DDI effect on linezolid.

The rifampin‐mediated induction of *ABCB1* was described using a maximum effect (*E*_*max*_) model, as described in Equations (3) and (4).

(3) $$\frac{\mathrm{dE}\left(t\right)}{\mathrm{dt}}=R_{\mathrm{syn},\mathrm{app}}-K_{\deg}\times E\left(t\right)$$

(4) $$R_{\mathrm{syn},\mathrm{app}}=R_{\mathrm{syn}}\times \left(1+\frac{E_{\max}\times \left[I\right]}{{\mathrm{EC}}_{50}+\left[I\right]}\right)$$

where *dE*(*t*)/*dt* is transporter turnover, *R*_*syn,app*_ denotes a zero‐order rate of transporter synthesis in the presence of an inducer, *K*_*deg*_ is the first‐order degradation rate constant, *E*(*t*) is the transporter concentration, *R*_*syn*_ is the rate of enzyme or transporter synthesis in the absence of inducer, *E*_*max*_ is the maximum induction effect in vivo, [*I*] is the free inducer concentration and *EC*_50_ is the rifampin concentration associated with producing half of the maximum effect in vivo.

The rifampin‐mediated inhibition of *ABCB1* and *ABCG2* was described using a competitive inhibition model as described in Equations (5) and (6).

(5) $$V=\frac{V_{\max}\times \left[S\right]}{K_{M,\mathrm{app}}+\left[S\right]}=\frac{K_{\mathrm{cat}}\times \left[E\right]\times \left[S\right]}{K_{M,\mathrm{app}}+\left[S\right]}$$

(6) $$K_{M,\mathrm{app}}=K_{M}\left(1+\frac{\left[I\right]}{K_{i}}\right)$$

In this model, *V* is the reaction velocity, *V*_*max*_ is the maximum reaction velocity, [*S*] is the free substrate concentration, *K*_*M,app*_ is the Michaelis–Menten constant in the presence of inhibitor, *K*_*cat*_ is the catalytic rate constant, [*E*] is the transporter concentration, *K*_*M*_ is the Michaelis–Menten constant in the absence of inhibitor, [*I*] is the free inhibitor concentration and *K*_*i*_ is the dissociation constant of the inhibitor‐enzyme complex.

Rifampin has been reported to inhibit *ABCG2* activity with a K_i_ of 9 μM.^29^ Additionally, rifampin induces the expression of *ABCG2* by 1.18‐fold to 2.7‐fold.^14^, ^15^, ^16^ In the current model, an average 1.5‐fold increase in *ABCG2* expression was implemented to reflect the induction effect of rifampin.

### 2.6 Simulations

#### 2.6.1 Linezolid PBPK model

The PBPK model was developed and evaluated using a built‐in PK‐SIM virtual population, with key demographic characteristics, such as, race, sex, age and weight, aligned with that reported in the clinical PK studies. Population simulations require information on age and weight range, so if ranges were not reported, individual simulations based on median or mean value were conducted instead of population simulations. The dose regimen (including administration route, dose amount and frequency) in the simulation was identical to that used in the clinical PK studies. For each PK arm, 10 times the number of virtual subjects relative to original subjects were simulated.

Human physiological parameters were derived from the default virtual population in PK‐Sim®. Relative tissue expression levels of enzymes and transporters were obtained from mRNA quantification by reverse transcription polymerase chain reaction, whereas their abundances were derived from proteomics measurements (pmol/mg protein) and scaled to the organ level using reported tissue protein yields^29^.

#### 2.6.2 Rifampin–linezolid DDI PBPK model

To elucidate the main transporters involved in the rifampin–linezolid interaction, we simulated the DDI under two scenarios; one assuming mediation by *ABCB1* alone and the other assuming combined involvement of *ABCB1* and *ABCG2*. The predicted PK data of linezolid with and without rifampin co‐administration were simulated and compared with observed data from previously published literature.^18^

### 2.7 Sensitivity analysis

In accordance with regulatory guidelines for the qualification and verification of PBPK platforms, it is essential to account for uncertainties in in‐vitro data, such as *K*_*i*_, *EC*_50_ and *E*_*max*_, to ensure the reliability of model predictions.^35^ Nilles et al. reported an *E*_*max*_ value of 5.1 and 7 after 96 and 120 h of rifampin exposure, respectively, with an *EC*_50_ of approximately 1 μM for *ABCB1* induction, and *K*_*i*_ value of 12.9 μM for *ABCB1* inhibition.^36^ Asaumi et al. reported a value of *E*_*max*_ (4), *EC*_50_ (0.0639 μM) for *ABCB1* induction and *K*_*i*_ (0.488 μM) for *ABCB1* inhibition.^37^ Two sets of published values were used to predict linezolid PK profiles in the sensitivity analysis.

### 2.8 PBPK model evaluation

#### 2.8.1 Linezolid PBPK model

The performance of the linezolid PBPK model was assessed by comparing the predicted and observed plasma concentration–time profiles, the fraction of drug excreted unchanged in urine (*Ae*), and the two main plasma exposure parameters (*AUC*, area under the concentration–time profile; and *C*_*max*_, peak concentration).

The mean relative deviation (MRD) was calculated to evaluate the predictive performance of plasma drug concentrations (Equation 7) while the geometric mean fold error (GMFE) was calculated to assess the predictive performance for PK parameters (*Ae*, *AUC* and *C*_*max*_) (Equation 8).

Additionally, *AUC* and *C*_*max*_ ratio, defined as the predicted value divided with the observed value were also calculated (Equations 9 and 10).

(7) $$\mathrm{MRD}=10^{x},\text{with}\;x=\sqrt{\;\frac{1}{m}\sum_{i=1}^{m}{\left(\log_{10}C_{\text{pred},i}-\log_{10}C_{\mathrm{obs},i}\right)}^{2}}$$

(8) $$\text{GMFE}=10^{x},\text{with}\;x=\frac{1}{n}\sum_{i=1}^{n}\;\left(\log_{10}{\mathrm{PK}}_{\text{pred},i}-\log_{10}{\mathrm{PK}}_{\mathrm{obs},i}\right)$$

(9) $$\mathrm{AUC}\;\text{ratio}=\frac{{\mathrm{AUC}}_{\text{pred}}}{{\mathrm{AUC}}_{\mathrm{obs}}}$$

(10) $$C_{\max}\text{ratio}=\frac{C_{\max,\text{pred}}}{C_{\max,\mathrm{obs}}}$$

where *C*_*pred,i*_ is the predicted plasma concentration, *C*_*obs,i*_ is the observed plasma concentration, *m* is the number of observed concentrations, *PK*_*pred,i*_ is the predicted PK parameter value (*Ae*, *AUC* or *C*_*max*_), *PK*_*obs,i*_ is the observed PK parameter value and *n* is the number of studies.

MRD and GMFE values ≤2 indicate adequate model performance. A prediction within a two‐fold error for *AUC* and *C*_*max*_ was considered acceptable whereas a prediction within a 1.5‐fold error was regarded as good predictive performance.

#### 2.8.2 Linezolid–rifampin DDI model

The performance of the DDI predictions were evaluated by calculating the exposure ratio (*AUC* and *C*_*max*_) of linezolid when given with RIF and alone, as shown in Equations (11) and (12).^38^

(11) $$\mathrm{DDI}\;\mathrm{AUC}\;\text{ratio}=\frac{{\mathrm{AUC}}_{\text{with}\;\mathrm{RIF}}}{{\mathrm{AUC}}_{\text{without}\;\mathrm{RIF}}}$$

(12) $$\mathrm{DDI}\;\text{Cmax ratio}=\frac{{\text{Cmax}}_{\text{with}\;\mathrm{RIF}}}{{\text{Cmax}}_{\text{without}\;\mathrm{RIF}}}$$

A predicted DDI ratio that fell within two‐fold of observed ratios were considered acceptable.^38^

The average fold error (*AFE*) and root mean square error (*RMSE*) were calculated to assess the bias and precision for the DDI prediction, expressed as Equations (13) and (14), where *n* denotes the number of studies.

(13) $$\mathrm{AFE}=10^{|\frac{1}{n}\sum \log\frac{\text{Predicted}\;\mathrm{DDI}\;\mathrm{AUC}\;\text{or}\;C_{\max}\;\text{ratios}\;}{\text{Observed}\;\mathrm{DDI}\;\mathrm{AUC}\;\text{or}\;C_{\max}\;\text{ratios}}|}$$

(14) $$\text{RMSE}=\sqrt{\frac{1}{n}\sum {\left(\text{Predicted}\;\mathrm{DDI}\;\mathrm{AUC}\;\text{or}\;C_{\max}\;\text{ratios}-\text{Observed}\;\mathrm{DDI}\;\mathrm{AUC}\;\text{or}\;C_{\max}\text{ratios}\right)}^{2}}$$

### 2.9 Prediction DDI between high dose rifampin and linezolid

Based on the developed rifampin–linezolid DDI PBPK model, the PK profile of linezolid in subjects co‐administered with standard and high doses of rifampin was predicted. Participants were assumed to receive rifampin once daily for seven consecutive days at dose levels of 20, 25, 30, 35 and 40 mg/kg. On Day 8, linezolid was administered orally at 600 twice a day for 7 days alongside rifampin. On Day 14, the PK profile of linezolid was predicted and the PK exposure parameters were summarized.

### 2.10 Nomenclature of targets and ligands

The drug and molecular target nomenclature conforms to the IUPHAR/BPS Guide to PHARMACOLOGY nomenclature classification. Key protein targets and ligands in this article are hyperlinked to corresponding entries online (http://www.guidetopharmacology.org) and are permanently archived in the ‘Concise guide to pharmacology 2021/22’.^39^

## 3 RESULTS

### 3.1 Linezolid PBPK model development and validation

**FIGURE 2:**
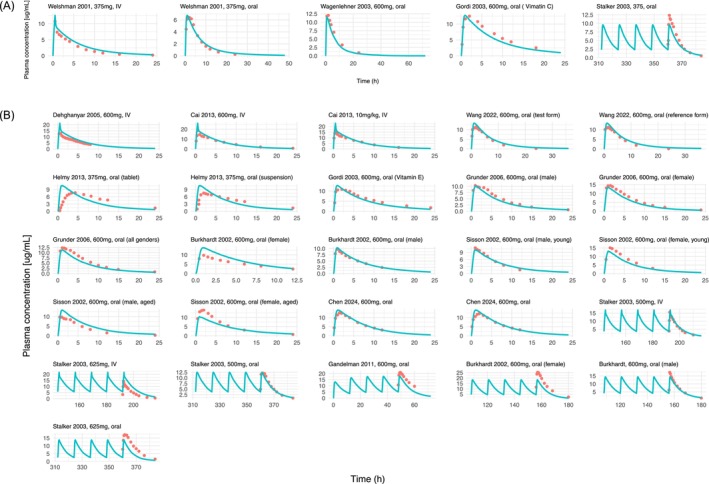
Predicted and observed plasma concentration–time profiles of linezolid in (A) training and (B) validation datasets in healthy volunteers. Solid lines represent model‐predicted mean values while circles indicate clinical observed mean values. The details on dosing regimens, sample sizes, subject demographics and references are provided in Table S1. The training dataset comprised 5 study arms from 4 PK studies, while the validation dataset included 26 PK arms from 11 studies that reported full PK profiles. The data presented by Chen et al. (2024) did not explicitly distinguish between the concentration–time profiles of the test and reference formulations.

**FIGURE 3:**
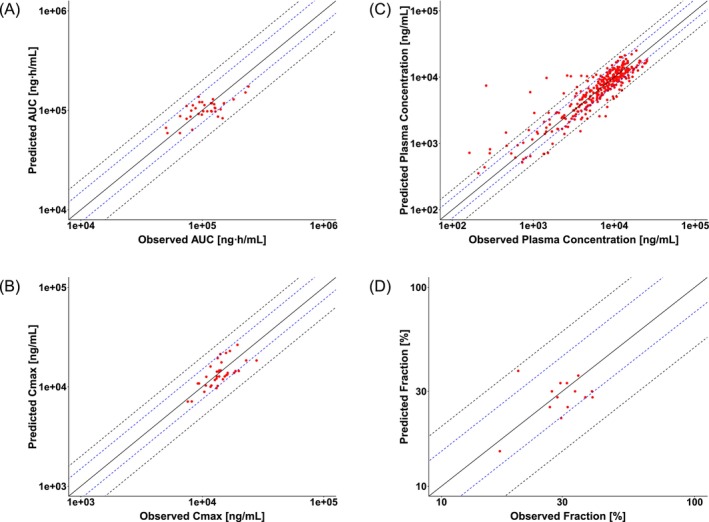
PBPK model predictions versus observed values of linezolid for (A) *AUC*_*inf*_, (B) *C*_*max*_, (C) plasma concentration and (D) fraction excreted unchanged in urine. The line of identity is presented as a solid line; 1.5‐fold dimensions and 2.0‐fold dimensions are shown using blue and black dashed lines, respectively.

The IV PBPK model for linezolid was developed successfully, with distribution (i.e., partition coefficients) and elimination parameters (*K*_*cat*_ values for *ABCB1* and *ABCG2*) optimized to improve model predictions. Subsequently, the IV model was extended to an oral PBPK model, where intestinal permeability was optimized to refine the absorption profile. The final PBPK model parameters for linezolid are summarized in Table 1. Figure 2A,B demonstrates a satisfactory predictive performance of the developed PBPK model in the training and validation dataset. Figure 3 depicts the correlation between predicted and observed *AUC*, *C*_*max*_, plasma concentrations and *Ae* of linezolid. The MRD for plasma concentrations was 1.51. All predicted *AUC* and *C*_*max*_ values fell within a two‐fold error of the observed values, with vast majority (34/36) falling within a 1.5‐fold error, as shown in Table S2. For *Ae*, only 1 out of 14 predictions exceeded the 1.5‐fold error (Table S3). The overall GMFE for *AUC*, *C*_*max*_ and *Ae* was 1.23, 1.21 and 1.21, respectively, indicating a high reliability and robustness across different evaluation metrics.

**TABLE 1:** The input parameters for the development of linezolid PBPK model.

Parameter	Value	Unit	Reference
Basic physio‐chemistry			
Molecular weight	337.35	g/mol	^40^
LogPo:w	0.55	—	^41^
Compound type	Monoprotic base	—	^18^
pKa	1.7	—	^18^, ^40^
Fraction unbound in plasma	69	%	^18^, ^40^
Solubility (in water)	3	g/L	^42^
Absorption			
Intestinal permeability	9 × 10^−6^ cm/min	cm/min	Optimized
Distribution			
Partition coefficients	Rowland and Rodger	—	—
Cellular permeabilities	PK‐Sim standards	—	—
Organ specified permeabilities	9 × 10^−4^ cm/min	cm/min	Optimized
Metabolism			
Plasma hepatic CL	0.65 for single‐dose model and 0.5 for multiple dose model	mL/min/kg	^5^
Transport and excretion			
*ABCB1*‐K_cat_	72	nmol/min/pmol transporter	Optimized
*ABCB1*‐Km	51	μmol/l	Optimized
*ABCG2*‐K_cat_	720	nmol/min/pmol transporter	Optimized
*ABCG2*‐Km	53	μmol/L	Optimized
*ABCB1* reference concentration	0.077	μmol/L	^29^
*ABCG2* reference concentration	0.025	μmol/L	^29^

### 3.2 Rifampin–linezolid DDI PBPK model

**FIGURE 4:**
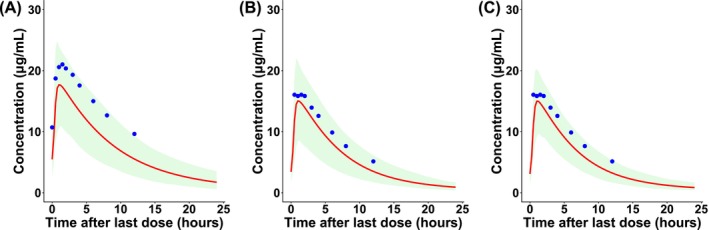
Plasma concentration–time profile of linezolid in healthy volunteers after (A) monotherapy, (B) rifampin‐mediated P‐glycoprotein (*ABCB1*) interaction and (C) rifampin‐mediated *ABCB1* and *ABCG2* interaction at steady state. Solid lines represent model‐predicted mean values while circles represent mean clinical observations. The shaded area represents the 95% prediction interval.

The linezolid PK profile was accurately predicted when co‐administrated with rifampin, as shown in Figure 4. The DDI ratio for *AUC*_0–12_ and *C*_*max*_ of linezolid were predicted under two different interaction mechanisms; *ABCB1*‐mediated interaction alone and in combination with *ABCG2*‐mediated interaction (Table 2). The predicted DDI ratios for AUC and *C*_*max*_ were 0.79 and 0.87, respectively, when considering *ABCB1*–mediated DDI alone, and 0.77 and 0.85, respectively, when the *ABCG2*–mediated DDI was also incorporated. The fold errors, expressed as *AFE*, for the predicted DDI ratio for *AUC* and *C*_*max*_ were 1.19 and 1.12, respectively, for *ABCB1* mediated DDI model and 1.16 and 1.10, respectively, for the combined *ABCB1*‐ and *ABCG2*‐mediated DDI model.

**TABLE 2:** Observed and predicted *AUC* and *C*_*max*_ of linezolid when administered as monotherapy and together with rifampin in healthy volunteers.

Index	Observed interaction			Predicted interaction (*ABCB1* mediated)			Predicted interaction (*ABCB1* and *ABCG2* mediated)		
	Linezolid	Linezolid + rifampin	Ratio	Linezolid	Linezolid + rifampin	Ratio	Linezolid	Linezolid + rifampin	Ratio
*AUC*_0–12_ (μg h/mL)	181.2	120.9	0.66	128.7	102.3	0.79	128.7	96.6	0.77
*C*_*max*_ (μg/mL)	23.0	17.9	0.77	18.4	16.1	0.87	18.4	15.7	0.85

*Note*: The model predicted *AUC* and *C*_*max*_ of linezolid with the assumption of either *ABCB1* or *ABCG2* and *ABCB1* mediated interaction. The kinetics parameters between rifampin versus *ABCB1* and *ABCG2* are from PK‐Sim model library.

Abbreviations: *AUC*, area under curve; *C*_*max*_, maximum concentration.

The RMSE was 0.13 and 0.1 for *AUC* and *C*_*max*_, for *ABCB1*‐mediated DDI alone, and 0.11 and 0.08 for *ABCB1*‐ and *ABCG2*‐mediated DDI.

Since the *ABCB1*‐ and *ABCG2*‐mediated interaction model showed lower bias in predicting the DDI ratio, it was selected as the final model for simulating linezolid PK in combination with high‐dose rifampicin.

Moreover, a sensitivity analysis was conducted using different *E*_*max*_, *EC*_50_ and *K*_*i*_ values taken from two published studies and showed only a minor difference in the predicted *AUC* and *C*_*max*_ ratios. In case of both *ABCG2*‐ and *ABCB1*‐mediated interaction, predicted *AUC* and *C*_*max*_ ratios had values of 0.67 and 0.79 from Nilles et al.^36^ and 0.73 and 0.78 from Asaumi et al.^37^

### 3.3 Prediction DDI between high‐dose rifampin and linezolid

**FIGURE 5:**
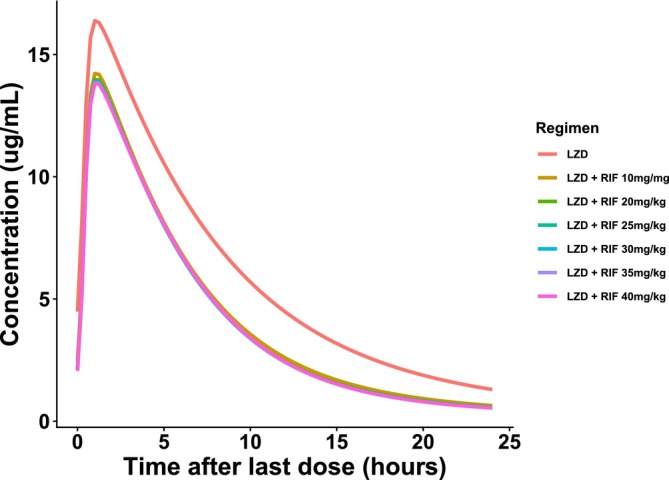
Simulated mean plasma concentration–time profiles of linezolid after oral administration of 600 mg, with or without co‐administration of rifampin at a standard dose (10 mg/kg/day) or higher doses of 20–40 mg/kg/day.

The PK parameters and profiles of linezolid administered alone or in combination with rifampin at standard or high doses are shown in Table 3 and Figure 5. At steady state, co‐administration of rifampin standard dose of 10 mg/kg reduced the AUC of linezolid by 30% and the *C*_*max*_ by 13%. Compared with the standard dose of rifampin, high doses of rifampin resulted in a similar reduction in *C*_*max*_ and *AUC* of linezolid.

**TABLE 3:** Predicted PK parameters of linezolid following oral administration of linezolid 600 twice a day alone or with concomitantly QD regimen of rifampin 10–40 mg/kg/day.

PK index	Unit	Linezolid	Linezolid + rifampin 10 mg/kg	Linezolid + rifampin 20 mg/kg	Linezolid + rifampin 25 mg/kg	Linezolid + rifampin 30 mg/kg	Linezolid + rifampin 35 mg/kg	Linezolid + rifampin 40 mg/kg
*AUC*_inf_	μg h/mL	161.5	113.6	110.0	109.2	108.9	108.8	108.8
*C*_*max*_	μg/mL	17.0	14.9	14.7	14.6	14.6	14.5	14.5

## 4 DISCUSSION

In the present study, a PBPK model was developed to predict DDI between rifampin and linezolid. Our findings demonstrate that *ABCB1* is the primary transporter mediating the interaction between the two drugs. Furthermore, the PBPK model predicted that a high dose of rifampin did not have an additional effect on the linezolid exposure compared with the standard dose of rifampin.

Transporters are believed to play a crucial role in the PK interaction between rifampin and linezolid. Previous studies suggest that *ABCB1* and *ABCG2* may be responsible for the observed DDI. However, the individual contributions and overall impact of these two transporters remain unclear. Hashimoto et al. previously reported a significant reduction in the *AUC*, *C*_*max*_ and bioavailability of orally administered linezolid in rat, following multiple doses of rifampin.^43^ This observation supports our hypothesis that the reduction in orally administered linezolid is due to the induction of intestinal *ABCB1* by rifampin.

A recent study confirmed that *ABCG2* functions as a transporter for linezolid.^6^ This finding raises important question regarding the potential role of *ABCG2* in linezolid PK and its involvement in the interaction with rifampin, given that rifampin has been reported to both inhibit and induce *ABCG2* expression.^13^, ^14^, ^15^, ^16^ In the current study, *ABCB1*‐ and *ABCG2*‐mediated model showed a slightly lower bias in prediction of DDI compared with that mediated by *ABCB1* alone. This suggests that *ABCG2* plays a role in the rifampin–linezolid interaction, but to a relatively minor extent. Several factors may explain this finding. First, *ABCG2* expression levels are significantly lower than those of *ABCB1*, as indicated by in vitro data.^29^ Second, although rifampin induces *ABCB1* expression by more than 3.5‐fold,^44^ its induction effect on *ABCG2* is relatively mild, ranging from 1.18‐fold to 2.7‐fold.^14^, ^15^, ^16^ These data suggest that rifampin‐induced upregulation of *ABCB1* is more pronounced than its effect on *ABCG2*.

High dose of rifampin has been reported to shorten the time to sputum culture conversion and also to be safe and well‐tolerated at doses up to 40 mg/kg.^45^ However, the potential for DDI between high doses of rifampin and linezolid remains underexplored. Previously, compared with the standard dose (10 mg/kg), a high dose of rifampin at 35 mg/kg was reported to have a substantial additional effect on the plasma exposure of dolutegravir, a substrate of *UGT1A1*, with a further 43% reduction in trough concentrations.^46^ Conversely, high‐dose of rifampin showed no additional effect on *CYP1A2*, mild additional induction of *CYP2C9*, *CYP2C19*, *CYP2D6* and *CYP3A*, but these changes were not clinically significant.^47^ For drugs that are *ABCB1* substrates, the interaction with rifampin is more complex, as rifampin is known to act as both an inducer at low concentration and an inhibitor of *ABCB1* at high concentrations.^12^ High concentrations of rifampin could potentially result in additional *ABCB1* inhibition, outweighing any induction, as observed in the interaction between high‐dose rifampin and digoxin, a sensitive *ABCB1* substrate.^47^ This study showed marginal inhibition of *ABCB1* at a 40 mg/kg dose, resulting in an additional 17% increase in PK exposure. In our PBPK model, a 40 mg/kg dose of rifampin produced a small further decrease in linezolid AUC, compared with standard 10 mg/kg dosing. This difference, compared with digoxin, could be partly explained by substrate sensitivity as digoxin is a more sensitive *ABCB1* substrate,^48^ compared with linezolid, resulting in an increase in PK exposure due to the additional inhibitory effect at high‐dose rifampin. However, for linezolid co‐administered with high‐dose rifampin, the induction effect appears to outweigh the inhibitory effect, resulting in a slight reduction in PK exposure.

A clinical study has previously evaluated the DDI between linezolid and clarithromycin, a known *ABCB1* inhibitor, thereby demonstrating the role of *ABCB1* in linezolid disposition.^49^ However, to date, no clinical investigation has assessed the DDI between linezolid and an *ABCG2* inhibitor, despite in vitro evidence suggesting that linezolid may serve as a substrate of *ABCG2*. Consequently, the relative contributions of *ABCB1* and *ABCG2* remain unclear in the absence of definitive clinical data. To address this knowledge gap, we conducted a sensitivity analysis by modifying the *K*_*cat*_ values of each transporter by 100%. The results revealed that alterations in *ABCB1 K*_*cat*_ exerted a 20‐ to 30‐fold greater effect on both *AUC* and *C*_*max*_ compared with changes in *ABCG2 K*_*cat*_, indicating that *ABCB1* plays a major role in linezolid pharmacokinetics, while the contribution of *ABCG2* appears to be minor.

Moreover, in our PBPK model, the removal of hepatic *ABCB1* expression is unlikely to affect the DDI results, as rifampin‐induced *ABCB1* expression in the liver is not expected to occur. Linezolid is not excreted unchanged in the faeces,^8^ and there is no evidence of involvement of hepatic efflux transporters. In addition, the primary mechanism of a PK interaction between rifampin and a *ABCB1* substrate is mainly due to intestinal *ABCB1* induction.^14^ Rifampin is primarily excreted as metabolites in bile,^15^ while only about 17% of the drug is recovered unchanged in urine.^16^ Rifampin itself is not recovered as parent drug in faeces or bile. Taken together, these findings suggest that hepatic *ABCB1* involvement or induction by rifampin is unlikely.

PBPK models for linezolid have been developed for various applications. Litjens et al.^6^ developed a PBPK model to accurately predict linezolid PK in plasma and CSF in both adult and paediatric patients with tuberculous meningitis, a severe form of tuberculosis, by incorporating several biological parameters related to critical illness and blood–brain barrier transporters. In the current study, PBPK model development and DDI assessment with rifampicin were conducted primarily in healthy subjects. The magnitude of the DDI may differ in the target patient population due to infection‐related changes in biological parameters. This warrants further investigation.

Additionally, the slight variation in *AUC* and *C*_*max*_ ratios observed with different in vitro values suggest a robust model and wide generalization of our results. PBPK models are increasingly recognized as essential tools for predicting DDIs and their application in transporter‐mediated interactions is also expanding.^50^ Given the complex nature of the rifampin–linezolid interaction, which involves multiple transporters such as *ABCB1*, a quantitative approach is necessary. The PBPK model developed in this study accurately predicted the transporter‐mediated DDI between rifampin and linezolid and can be used to extrapolate the DDI effect at different doses.

Our study has several limitations. First, we assumed that linezolid's non‐renal clearance occurs solely through hepatic metabolism. A more comprehensive PBPK model accounting for non‐renal clearance mechanisms can further understand linezolid pharmacokinetics in human. Second, the *K*_*m*_ value for *ABCB1* and *ABCG2* were not reported in the literature; therefore, they were initially assumed to be equal to the incubation concentrations and subsequently optimized during model development. Third, the verification of the DDI model relied on data from a single clinical trial,^18^ which may introduce uncertainty.

## 5 CONCLUSIONS

The PBPK modelling analysis indicated that both *ABCG2* and *ABCB1* contributed to the DDI between linezolid and rifampin, with *ABCB1* playing the predominant role in the interaction. Furthermore, linezolid co‐administration with either a standard or high dose of rifampin resulted in a similar reduction in linezolid plasma exposure.

## AUTHOR CONTRIBUTIONS

H. D. N., J. D. and J. T. designed the research. H. D. N. and J. D. performed the research. H. D. N. and J. D. analysed the data. H. D. N., V. H. P. and J. D. wrote the manuscript. R. M. H. and J. T. reviewed the manuscript.

## CONFLICT OF INTEREST STATEMENT

The authors have no conflicts of interest to declare.

## Supporting information


**Table S1:** List of clinical studies used for the development and evaluation of the PBPK model of linezolid.
**Table S2**: Clinical observed and predicted PK parameters of linezolid simulated by the developed PBPK.
**Table S3**: Clinical observed and predicted fraction of linezolid excreted unchanged in the urine.

## Data Availability

The datasets generated during and/or analysed during the current study are available from the corresponding author on reasonable request.
