# Supplementary material for: Assessing transporter‐mediated rifampin–linezolid interaction using physiologically‐based pharmacokinetic modelling
Source: Br J Clin Pharmacol. 2026 Jan 26;92(6):1845–56. doi: 10.1002/bcp.70443 (PMC13206452; doi:10.1002/bcp.70443)
Supplement: Supplementary file 1 — Table S1: List of clinical studies used for the development and evaluation of the PBPK model of linezolid. Table S2: Clinical observed and predicted PK parameters of linezolid simulated by the developed PBPK. Table S3: Clinical observed and predicted fraction of linezolid excreted unchanged in the urine. [file BCP-92-1845-s001.docx]

**SUPPLEMENTARY MATERIAL**

**Assessing Transporter-Mediated Rifampin–Linezolid Interaction Using Physiologically-Based Pharmacokinetic Modeling**

*H.D. Nguyen, V.H. Pham, R.M. Hoglund, J. Tarning, J. Ding*

**Tables and Figures**

Table S1. List of clinical studies used for the development and evaluation of the PBPK model of linezolid

Table S2. Clinical observed and predicted PK parameters of linezolid simulated by the developed PBPK model

Table S3. Clinical observed and predicted fraction of linezolid excreted unchanged in the urine

**Supplementary Reference**

**Table S1.** List of clinical studies used for the development and evaluation of the PBPK model of linezolid

| **Study** | **Sample size** | **Ethnicity** | **Age (years)** | **Female (%)** | **Weight (kg)** | **Height (cm)** | **Route** | **Dose (mg)** | **Regimen** | **Assay** |
| --- | --- | --- | --- | --- | --- | --- | --- | --- | --- | --- |
| Training | | | | | | | | | | |
| Welshman 2001(1) | 12 | Caucasian | 39 (25–53) | 41 | 72 (53–86) | 168 (162-176) | IV | 375 | S | HPLC |
| Welshman 2001 (1) | 12 | Caucasian | 39 (25–53) | 41 | 72 (53–86) | 168 (162-176) | oral | 375 | S | HPLC |
| Wagenlehner 2003 (2) | 12 | Caucasian | 27 (19 - 42) | 50 | 68 (58 - 87) | 171 (159 -187) | oral | 600 | S | LC-MS/MS |
| Gordi 2003 (3) | 14 | Chinese, Indian | 24 | 0.07 | 71 | NA | oral | 600 | S | HPLC/MS/MS |
| Stalker 2003 (4) | 6 | Caucasian | 38 (26–53) | 33 | 66 (54–84) | 167 (155-177) | oral | 375 | M | HPLC |
| Validation | | | | | | | | | | |
| Stalker 2003 (4) | 6 | Caucasian | 29 (19-54) | 16.6 | 75 (61–83) | 178 (173-185) | IV | 500 | S | HPLC |
| Dehghanyar 2005 (5) | 10 | Caucasian | 55 ±10 | 50 | 66 ± 8 | 168 | IV | 600 | S | HPLC |
| Cai 2013 (6) | 10 | Chinese | 25.8 | 0 | 52.8 | NA | IV | 600 | S | LC-MS |
| Stalker 2003 (4) | 6 | Caucasian | 25 (19–33) | 0 | 79 (68–95) | 181 (168-191) | IV | 625 | S | HPLC |
| Cai 2013 (6) | 10 | Chinese | 25.8 | 0 | 52.8 | NA | IV | 10 mg/kg | S | LC-MS |
| Stalker 2003 (4) | 6 | Caucasian | 28 (19–39) | 33 | 71 (61–95) | 176(168–185) | oral | 500 | S | HPLC |
| Wang 2022 (7) | 24 | Chinese | 31 (19-50) | 33 | 65 (47- 77) | 168 (152- 186) | oral | 600 | S | LC-MS/MS |
| Helmy 2013 (8) | 28 | Egypt | 23 (20–35) | 0 | 73 (63–84) | NA | oral | 600 | S | HPLC |
| Gordi 2003 (3) | 14 | Chinese, other | 24 | 0 | 71 | NA | oral | 600 | S | HPLC/MS/MS |
| Grunder 2006 (9) | 9 | Caucasian | 30 (21–47) | 0 | 78 (69–87) | 181 (175-184) | oral | 600 | S | HPLC |
| Grunder 2006 (9) | 9 | Caucasian | 24 (22–25) | 100 | 66 (53–74) | 171 (164-177) | oral | 600 | S | HPLC |
| Grunder 2006 (9) | 18 | Caucasian | 27 (21–47) | 50 | 72.2 | 175 (164-184) | oral | 600 | S | HPLC |
| Burkhardt 2002 (10) | 6 | Caucasian | 32.2 | 100 | 65.2 | 167 | oral | 600 | S | HPLC |
| Burkhardt 2002 (10) | 6 | Caucasian | 3 | 0 | 82.2 | 182 | oral | 600 | S | HPLC |
| Sisson 2002 (11) | 8 | Caucasian | 30 (22–38) | 0 | 78 (65–94) | 177 (169-184) | oral | 600 | S | HPLC |
| Sisson 2002 (11) | 7 | Caucasian | 30 (21–38) | 100 | 61(55–71) | 164 (155-171) | oral | 600 | S | HPLC |
| Sisson 2002 (11) | 6 | Caucasian | 70 (65–75) | 0 | 87(78–94) | 180 (174-185) | oral | 600 | S | HPLC |
| Sisson 2002 (11) | 8 | Caucasian | 70 (66–75) | 100 | 70 (59–83) | 164 (158-170) | oral | 600 | S | HPLC |
| Chen 2024 (12) | 24 | Chinese | 26(20- 36) | 58 | 63 (53-78) | 167 (153- 186) | oral | 600 | S | LC-MS/MS |
| Stalker 2003 (4) | 6 | Caucasian | 33 (22–48) | 33 | 73 (54–81) | 173 (152-189) | oral | 625 | S | HPLC |
| Stalker 2003 (4) | 6 | Caucasian | 29 (19–54) | 16.6 | 75 (61–83) | 178 (173-185) | IV | 500 | M | HPLC |
| Stalker 2003 (4) | 6 | Caucasian | 25 (19–33) | 0 | 79 (68–95) | 181(168–191) | IV | 625 | M | HPLC |
| Stalker 2003 (4) | 6 | Caucasian | 28 (19–39) | 33 | 73 (61–95) | 176 (168-185) | oral | 500 | M | HPLC |
| Gandelman 2011 (13) | 16 | East Asian | 28.6 (21-41) | 0 | 71 (56.0-90.0) | NA | oral | 600 | M | LC-MS/MS |
| Burkhardt 2002 (10) | 6 | Caucasian | 32 | 100 | 65 | 167 | oral | 600 | M | HPLC |
| Burkhardt 2002 (10) | 6 | Caucasian | 31 | 0 | 82 | 182 | oral | 600 | M | HPLC |
| Stalker 2003 (4) | 6 | Caucasian | 33 (22–48) | 33 | 73 (54–81) | 173 (152-189) | oral | 625 | M | HPLC |

NA: not available. Regimen S: single dose regimen. Regimen M: multiple dose regimen; HPLC: high-performance liquid chromatography; LC-MS: liquid chromatography-mass spectrometry; LC-MS/MS: liquid chromatography-tandem mass spectrometry.

**Table S2**. Clinical observed and predicted PK parameters of linezolid simulated by the developed PBPK model

| **Study** | **Route** | **Dose (mg)** | **Regimen** | **C_max_ (μg/mL)** | | | **AUC (μg•h/mL)** | | |
| --- | --- | --- | --- | --- | --- | --- | --- | --- | --- |
|  |  |  |  | **Observed** | **Predicted** | **Ratio** | **Observed** | **Predicted** | **Ratio** |
| ***Training*** | | | | | | | | | |
| Welshman 2001(1) | IV | 375 | S | 10.8 | 12.6 | 1.17 | 50.3 | 66.6 | 1.32 |
| Welshman 2001 (1) | oral | 375 | S | 7.6 | 7.1 | 0.93 | 51.7 | 59.0 | 1.14 |
| Wagenlehner 2003 (2) | oral | 600 | S | 13.1 | 12.1 | 0.92 | 118.6 | 98.4 | 0.83 |
| Gordi 2003 (3) | oral | 600 | S | 13.8 | 12.7 | 0.92 | 151.2 | 117.9 | 0.78 |
| Stalker 2003 (4) | oral | 375 | M | 13.1 | 9.7 | 0.74 | 82.8 | 63.7 | 0.77 |
| ***Validation*** | | | | | | | | | |
| Stalker 2003 (4) | IV | 500 | S | 11.7 | 16 | 1.37 | 65.8 | 87.3 | 1.33 |
| Dehghanyar 2005 (5) | IV | 600 | S | 14.1 | 21.3 | 1.51 | 88.1 | 104.9 | 1.19 |
| Cai 2013 (6) | IV | 600 | S | 19.5 | 26.4 | 1.35 | 122.5 | 129.3 | 1.06 |
| Stalker 2003 (4) | IV | 625 | S | 13.4 | 19.4 | 1.45 | 83.6 | 112,2 | 1.34 |
| Cai 2013 (6) | IV | 10 mg/kg | S | 16.9 | 22.9 | 1.36 | 104.3 | 112.8 | 1.08 |
| Stalker 2003 (4) | oral | 375 | S | 8.2 | 7.12 | 0.87 | 65.5 | 59.0 | 0.90 |
| Stalker 2003 (4) | oral | 500 | S | 10.4 | 8.9 | 0.86 | 74.3 | 82.2 | 1.11 |
| Wang 2022 (7) | oral | 600 | S | 13.7 | 14.2 | 1.04 | 99.8 | 120.9 | 1.21 |
| Wang 2022 (7) | oral | 600 | S | 13.2 | 14.2 | 1.08 | 94.8 | 120.9 | 1.28 |
| Helmy 2013 (8) | oral | 600 | S | 9.4 | 10.8 | 1.15 | 119.6 | 97.3 | 0.81 |
| Helmy 2013 (8) | oral | 600 | S | 9.2 | 10.8 | 1.17 | 128.4 | 97.3 | 0.76 |
| Gordi 2003 (3) | oral | 600 | S | 13.2 | 12.7 | 0.96 | 150.7 | 117.1 | 0.78 |
| Grunder 2006 (9) | oral | 600 | S | 13.4 | 10.2 | 0.76 | 93.3 | 92.0 | 0.99 |
| Grunder 2006 (9) | oral | 600 | S | 16.2 | 13.8 | 0.85 | 126.7 | 105.9 | 0.84 |
| Grunder 2006 (9) | oral | 600 | S | 14.8 | 11.9 | 0.80 | 110 | 97.7 | 0.89 |
| Burkhardt 2002 (10) | oral | 600 | S | 18.3 | 13.8 | 0.75 | 141.6 | 102.3 | 0.72 |
| Burkhardt 2002 (10) | oral | 600 | S | 10.7 | 10.3 | 0.96 | 134.7 | 86.4 | 0.64 |
| Sisson 2002 (11) | oral | 600 | S | 11.7 | 10.0 | 0.85 | 80 | 86.4 | 1.08 |
| Sisson 2002 (11) | oral | 600 | S | 16.1 | 13.3 | 0.83 | 128 | 105.1 | 0.82 |
| Sisson 2002 (11) | oral | 600 | S | 11.9 | 10.3 | 0.87 | 74.3 | 110.6 | 1.49 |
| Sisson 2002 (11) | oral | 600 | S | 15.8 | 13.1 | 0.83 | 125.4 | 115.0 | 0.92 |
| Chen 2024 (12) | oral | 600 | S | 14.2 | 14.6 | 1.03 | 118 | 117.8 | 1.00 |
| Chen 2024 (12) | oral | 600 | S | 13.8 | 14.6 | 1.06 | 120 | 117.8 | 0.98 |
| Stalker 2003 (4) | oral | 625 | S | 12.7 | 11.7 | 0.92 | 102 | 97.8 | 0.96 |
| Stalker 2003 (4) | IV | 500 | M | 14.4 | 17.6 | 1.22 | 81.2 | 99.3 | 1.22 |
| Stalker 2003 (4) | IV | 625 | M | 15.7 | 21.9 | 1.39 | 93.4 | 136.8 | 1.46 |
| Stalker 2003 (4) | oral | 500 | M | 15.3 | 12.7 | 0.83 | 99.2 | 90.4 | 0.91 |
| Gandelman 2011 (13) | oral | 600 | M | 23 | 18.4 | 0.80 | 181.2 | 128.7 | 0.71 |
| Burkhardt 2002 (10) | oral | 600 | M | 28 | 18.4 | 0.66 | 240 | 173.5 | 0.72 |
| Burkhardt 2002 (10) | oral | 600 | M | 20 | 14.4 | 0.72 | 227 | 151.2 | 0.67 |
| Stalker 2003 (4) | oral | 625 | M | 18.8 | 14.4 | 0.77 | 147 | 83.2 | 0.57 |

**Table S3**. Clinical observed and predicted fraction of linezolid excreted unchanged in the urine

| **Study ID** | **Collection Time (h)** | **Observed Fraction (%)** | **Predicted Fraction (%)** | **Ratio** |
| --- | --- | --- | --- | --- |
| ***Training*** | | | | |
| Wagenlehner 2003 (2) | 6 | 17 | 15 | 0.88 |
| Wagenlehner 2003 (2) | 12 | 29.7 | 22 | 0.74 |
| Wagenlehner 2003 (2) | 24 | 37 | 28 | 0.76 |
| Wagenlehner 2003 (2) | 48 | 39.3 | 30 | 0.76 |
| ***Validation*** | | | | |
| Welshman 2001 (1) | 48 | 34.7 | 36 | 1.04 |
| Welshman 2001 (1) | 48 | 29.5 | 33 | 1.12 |
| Gordi 2003 (3) | 24 | 39.5 | 28 | 0.71 |
| Gordi 2003 (3) | 24 | 28.7 | 28 | 0.98 |
| Burkhardt 2002 (10) | 12 | 31.5 | 25 | 0.79 |
| Burkhardt 2002 (10) | 12 | 26.8 | 25 | 0.93 |
| Sisson 2002 (11) | 48 | 27.3 | 30 | 1.10 |
| Sisson 2002 (11) | 48 | 33.7 | 30 | 0.89 |
| Sisson 2002 (11) | 48 | 20.1 | 38 | 1.89 |
| Sisson 2002 (11) | 48 | 31.3 | 33 | 1.05 |

**Supplementary Reference**

1. Welshman IR, Sisson TA, Jungbluth GL, Stalker DJ, Hopkins NK. Linezolid absolute bioavailability and the effect of food on oral bioavailability. Biopharmaceutics & drug disposition. 2001;22(3):91-7.

2. Wagenlehner FM, Wydra S, Onda H, Kinzig-Schippers M, Sörgel F, Naber KG. Concentrations in plasma, urinary excretion, and bactericidal activity of linezolid (600 milligrams) versus those of ciprofloxacin (500 milligrams) in healthy volunteers receiving a single oral dose. Antimicrobial agents and chemotherapy. 2003;47(12):3789-94.

3. Gordi T, Tan LH, Hong C, Hopkinsy NJ, Francom SF, Slatter JG, et al. The pharmacokinetics of linezolid are not affected by concomitant intake of the antioxidant vitamins C and E. The Journal of Clinical Pharmacology. 2003;43(10):1161-7.

4. Stalker DJ, Jungbluth GL, Hopkins NK, Batts DH. Pharmacokinetics and tolerance of single-and multiple-dose oral or intravenous linezolid, an oxazolidinone antibiotic, in healthy volunteers. Journal of Antimicrobial Chemotherapy. 2003;51(5):1239-46.

5. Dehghanyar P, Bürger C, Zeitlinger M, Islinger F, Kovar F, Müller M, et al. Penetration of linezolid into soft tissues of healthy volunteers after single and multiple doses. Antimicrobial agents and chemotherapy. 2005;49(6):2367-71.

6. Cai Y, Chai D, Falagas ME, Karageorgopoulos DE, Wang R, Bai N, et al. Weight-adjusted versus fixed dose of linezolid for Chinese healthy volunteers of higher and lower body weight: a phase I pharmacokinetic and pharmacodynamic study. Expert Opinion on Investigational Drugs. 2013;22(3):309-15.

7. Wang S, Guo S, Han Y, Ni S, Wu F, Dai Y, et al. Bioequivalence of Two Linezolid Tablets Under Fed and Fasting Conditions in Healthy Chinese Individuals. Clinical Pharmacology in Drug Development. 2022;11(12):1481-8.

8. Helmy S. Pharmacokinetics and relative bioavailability evaluation of linezolid suspension and tablet formulations. Drug research. 2013;63(09):489-94.

9. Grunder G, Zysset-Aschmann Y, Vollenweider F, Maier T, Krähenbühl S, Drewe J. Lack of pharmacokinetic interaction between linezolid and antacid in healthy volunteers. Antimicrobial agents and chemotherapy. 2006;50(1):68-72.

10. Burkhardt O, Borner K, von der Höh N, Köppe P, Pletz MW, Nord CE, et al. Single-and multiple-dose pharmacokinetics of linezolid and co-amoxiclav in healthy human volunteers. Journal of Antimicrobial Chemotherapy. 2002;50(5):707-12.

11. Sisson LT, Jungbluth G, Hopkins N. Age and sex effects on the pharmacokinetics of linezolid. European journal of clinical pharmacology. 2002;57:793-7.

12. Chen H, Xu H, Yuan F, Li H, Sheng L, Liu C, et al. Pharmacokinetics and Safety of Linezolid Tablets of 2 Different Manufacturers in Healthy Chinese Subjects in Fasting and Fed States. Clinical Pharmacology in Drug Development. 2024;13(11):1239-44.

13. Gandelman K, Zhu T, Fahmi OA, Glue P, Lian K, Obach RS, et al. Unexpected effect of rifampin on the pharmacokinetics of linezolid: in silico and in vitro approaches to explain its mechanism. The Journal of Clinical Pharmacology. 2011;51(2):229-36.
